# Optimizing antidotal treatment with the oral HSP90 inhibitor TAS-116 against hydrochloric acid-induced pulmonary fibrosis in mice

**DOI:** 10.3389/fphar.2022.1034464

**Published:** 2022-11-07

**Authors:** Pavel A. Solopov, Ruben Manuel Luciano Colunga Biancatelli, Christiana Dimitropolou, Tierney Day, John D. Catravas

**Affiliations:** ^1^ Frank Reidy Research Center for Bioelectrics, Old Dominion University, Norfolk, VA, United States; ^2^ School of Medical Diagnostic & Translational Sciences, College of Health Sciences, Old Dominion University, Norfolk, VA, United States

**Keywords:** idiopathic pulmonarry fibrosis, Hsp90 inhibitior, TAS-116, mice, hydrochloric acid

## Abstract

Exposure to high concentrations of hydrochloric acid (HCl) can lead to severe acute and chronic lung injury. In the aftermath of accidental spills, victims may be treated for the acute symptoms, but the chronic injury is often overlooked. We have developed a mouse model of acute and chronic lung injury, in which the peak of acute lung injury occurs on the day 4 after HCl exposure. We have also demonstrated that HSP90 inhibitors are effective antidotes when administered starting 24 h after HCl. In this study we examined the hypothesis that the novel oral HSP90 inhibitor TAS-116 can effectively ameliorate HCl-induced lung injury even when treatment starts at the peak of the acute injury, as late as 96 h after HCl. C57BI/6J mice were intratracheally instilled with 0.1N HCl. After 24 or 96 h, TAS-116 treatment began (3.5, 7 or 14 mg/kg, 5 times per week, p. o.) for either 2,3 or 4 or weeks. TAS-116 moderated the HCl-induced alveolar inflammation, as reflected in the reduction of white blood cells and total protein content in bronchoalveolar lavage fluid (BALF), overexpression of NLRP3 inflammasome, and inhibited the activation of pro-fibrotic pathways. Furthermore, TAS-116 normalized lung mechanics and decreased the deposition of extracellular matrix proteins in the lungs of mice exposed to HCl. Delayed and shortened treatment with TAS-116, successfully blocked the adverse chronic effects associated with acute exposure to HCl.

## 1 Introduction

HCl is one of the most used industrial chemical compounds in the world with numerous applications in oil and gas production, as a cleaning agent, and in the steel and leather industries. Accidental spills of HCl occur every year in industrial settings or during transportation. Acute exposure to high concentrations can cause severe acute and chronic, potentially fatal, lung injury (Occupational exposures to mists and, 1992). The National Research Council concluded that exposure to HCl results in pain, coughing, inflammation, edema and desquamation in the upper respiratory tract. High concentrations might also produce constriction of the larynx and bronchi and closure of the glottis (National Research Council (US) Committee on Acute Exposure Guideline Levels, 2010). A clinical study involving 170 fire fighters identified hydrogen chloride as an important contributor to respiratory symptoms and one fatal case with hemorrhage, edema, and inflammation of the lungs was reported (Dyer and Esch, 1976). Since in some cases symptoms appeared 2-to 3 days after the incident (Boyce and Simpson, 1996), the victims often do not seek medical attention immediately after exposure.

Heat shock protein 90 (HSP90) is an ATP-dependent molecular chaperone that is present in procaryotic and eucaryotic cells and facilitates the maturation of clients (kinases, transcription factors, signaling proteins, steroid hormone receptors, and numerous other proteins) that are involved in various cellular pathways (Schopf et al., 2017). Inhibition of HSP90 transforms the HSP90-client protein complex, leading to reduced activity, misfolding, ubiquitination and proteasomal degradation of client proteins (Butler et al., 2015). Association with a multiplicity of signaling pathways has positioned HSP90 as a promising target for cancer treatment. Recent studies suggest that HSP90 inhibitors may have antifibrotic properties (Sontake et al., 2017; Sanchez et al., 2020). A wealth of epidemiological studies demonstrates a strong correlation between organ fibrosis and cancer (Landolt et al., 2020). Experimental and clinical investigations suggest that these diseases have overlapping properties, such as activation of fibroblasts and growth factors, progressive extracellular matrix (ECM) deposition and similar pathogenic pathways (Piersma et al., 2020).

In previous studies we reported that 4-week treatment with HSP90 inhibitors (AUY-922, i. p. or AT13387, s. c.) administered starting 24 h after exposure to 0.1N HCl, successfully prevented chronic lung injury and pulmonary fibrosis in mice (Marinova et al., 2020; Colunga Biancatelli et al., 2022a). 4-(1H-pyrazolo[3,4-b] pyridine-1-yl) benzamide, or TAS-116, also known as Pimitespib, is a highly selective inhibitor of heat shock protein 90α and β, -with null activity on GRP94 or TRAP-1- that demonstrates potent antitumor activity and minimal toxicity compared to earlier generations of HSP90 inhibitors (Ohkubo et al., 2015). TAS-116 is structurally distinct from other HSP90 inhibitors and exhibits less hepatotoxicity and lacks the ability to penetrate the blood brain barrier (Doi et al., 2019). Moreover, oral bioavailability of this inhibitor allows for a more flexible dosing schedule compared with parenteral administration. In this study, we investigated the optimal dosing and windows of administration of TAS-116 against HCl-induced pulmonary fibrosis and chronic lung injury in mice.

## 2 Materials and methods

### 2.1 Materials

TAS-116 (Pimitespib) was purchased from MedChemExpress, HCl (37%), ACS grade, methacholine chloride USP grade, radioimmunoprecipitation assay (RIPA) buffer, and protease inhibitor cocktail were supplied from Sigma-Aldrich Corporation (St. Louis, MO, United States). Socumb (pentobarbital sodium) USP grade, AnaSed (xylazine) USP grade, and Ketaset (ketamine) USP grade were obtained by Henry Schein Animal Health (Pittsburg, PA, United States). Formaldehyde ACS reagent, 37 wt%, was purchased from ThermoFisher Scientific (Waltham, MA, United States), the BCA Protein assay kit from Pierce Co. (Rockford, IL, United States), EDTA and Amersham Protran 0.45 μm nitrocellulose blotting membranes from GE Healthcare (Chicago, IL, United States), TRIzol and SuperScript VILO reverse transcriptase kit from Invitrogen (Carlsbad, CA, United States), RNeasy Mini Kit from Qiagen (Hilden, Germany), and SYBR Green Master Mix from Applied Biosystems (Carlsbad, CA, United States). All primers used for real-time quantitative PCR were purchased from Integrated DNA Technologies, Inc. (Coralville, IA, United States). SDS-PAGE, ProtoGel (30% acrylamide mix), and TEMED were from National Diagnostics (Atlanta, GA, United States), Tris-HCl buffer from Teknova (Hollister, CA, United States), 10% sodium dodecyl sulfate (SDS) and ammonium persulfate (APS) from ThermoFisher Scientific, and Protein Dual Color Standards and Tricine Sample Buffer from Bio-Rad Laboratories (Hercules, CA, United States). For antibodies used in Western blotting, rabbit total and phosphorylated MAPK ERK 44-42 (Thr202/Tyr204), rabbit total and phosphorylated SMAD2 (Ser423/425), NLRP3 inflammasome antibodies were obtained from Cell Signaling Technology, Inc. (Danvers, MA, United States), mouse HSP90, rabbit Phospho-HSP90 (Ser226), Collagen Type I A2 and Mouse/Human TGF-β1 ELISA Kit were purchased from ThermoFisher Scientific. Mouse monoclonal β-actin antibody from Sigma-Aldrich Corporation (dilution 1:1000), and IRDye 800CW Goat anti-rabbit and IRDye 680RD Goat anti-mouse antibodies (dilution 1:5000) from LI-COR Biosciences (Lincoln, NE, United States).

### 2.2 Ethical statement

Animal studies were approved by the Institutional Animal Care and Use Committee (IACUC) of Old Dominion University (Protocol #19-014), abide by the principles of animal experimentation as published by the American Physiological Society, and were carried out in Animal Biosafety Level 2 (ABSL-2) facility at the Frank Reidy Research Center for Bioelectrics, ODU, Norfolk, VA.

### 2.3 Animals and treatment groups

Male C57Bl/6J mice, obtained from Jackson Laboratories (Bar Harbor, ME, United States), 8–10 weeks old, 23–26 g body weight, were housed in pathogen-free facility. Animals were intratracheally instilled with vehicle (normal saline) or HCl (0.1 N) and treated after 24 h with either vehicle (10% DMSO in corn oil) or with the HSP90 inhibitor TAS-116 (3.5, 7 or 14 mg/kg). Mice were randomly divided into six treatment groups: 1) mice that were exposed to normal saline (VEH); 2) mice that were exposed to 0.1N HCl and treated with vehicle (10% DMSO in corn oil) 5 times/week p. o. *via* gavage needle (HCl); (Wong et al., 2018) mice that were exposed to 0.1N HCl and treated orally with TAS-116 7 mg/kg 5 x/week starting 24 h post-instillation for 4 weeks (TAS 24 h 4w); 4) mice that were exposed to 0.1N HCl and treated orally with TAS-116, 7 mg/kg, 5 times/week starting 96 h post-instillation for 4 weeks (TAS 96 h 4w); 5) mice that were exposed to 0.1N HCl and treated orally with TAS-116 7 mg/kg 5 x/week starting 96 h post-instillation for 3 weeks (TAS 96 h 3w); 6) mice that were exposed to 0.1N HCl and treated orally with TAS-116 7 mg/kg 5 times/week starting 96 h post-instillation for 2 weeks (TAS 96 h 2w). On day 0, mice were anesthetized with intraperitoneal (i.p.) injections of xylazine (6 mg/kg) and ketamine (60 mg/kg). An i. p. bolus of sterile saline (10 μl/g) was given as pre-emptive fluid resuscitation. The surgical field was cleaned and disinfected with Betadine and 70% alcohol, a 1 cm neck skin incision and blunt dissection of salivary glands were made to visualize the trachea. Mice were suspended in upright position from their incisors and a fine, (20G) plastic i. v. catheter was inserted into the trachea (∼1.5 cm) in such a way that it could be seen through the rings of the trachea. Freshly prepared 0.1 N hydrochloric acid (groups 2–6) or sterile saline (group 1) was instilled (2 μL/g body weight) and flushed with 100 µl air. The catheter was withdrawn, the neck incision closed by the surgical adhesive, and the animals were placed in the ventral position in a small chamber with oxygen and observed constantly for the next 4 h for signs of respiratory distress. Mice were returned to their cages and monitored first day hourly and then daily for abnormal physical appearances. All analyses were performed at 30 days post instillation of HCl.

### 2.4 Bronchoalveolar lavage fluid white blood cell number and total protein concentration

BALF was obtained by instilling and withdrawing sterile 1 x PBS (1 ml) *via* a tracheal cannula, as we previously described (Solopov et al., 2020). The total number of leukocytes was counted using a hemocytometer. After the BALF was centrifuged at 2500 × *g* for 10 min, the supernatant was collected for total protein analysis. The protein concentration was estimated using a bicinchoninic acid (BCA) Protein Assay Kit according to manufacturer’s protocol. BALF supernatant TGF-β1 was analyzed in triplicate *via* a mouse/human TGF-β1 coated ELISA kit.

### 2.5 Histopathology, immunohistochemistry and lung fibrosis scoring

Mice were euthanized, the chest was opened and the lungs were fixed with formalin and embedded in paraffin, as we previously described (Solopov et al., 2020). Sections 5 µm thick were prepared from the blocks and stained with Masson’s trichrome and for Inflammasome NLR Family Pyrin Domain-Containing Protein 3 (NLRP3) at a dilution of 1:1280. Twenty randomly selected fields from each slide were examined under ×20 and ×40 magnifications. All the trichrome-stained slides were scored by the Ashcroft scoring scale to estimate the severity of pulmonary fibrosis (Ashcroft et al., 1988) by an investigator blinded to the identity of the animal groups.

### 2.6 Lung tissue collection

Immediately after euthanasia, the lungs were dissected from the thorax, snap-frozen, and prepared for subsequent analysis as we previously described (Solopov et al., 2020).

### 2.7 Western blot analysis

Proteins in lung tissue homogenates were extracted from snap-frozen lungs by sonication in ice-cold RIPA buffer with added protease inhibitor cocktail (100:1). The protein lysates were mixed under slow agitation for 3 h at 4°C, and then centrifuged twice at 14000× *g* for 10 min. The supernatants were gently aspirated, and total protein concentration was measured using the micro-BCA assay. The samples were first mixed with Tricine Sample Buffer, then heated up to 95°C for 10 min, then separated on a 10% polyacrylamide SDS gel by electrophoresis. Proteins were then transferred from the gel to a nitrocellulose membrane and incubated overnight with the appropriate primary antibody (1:1000), followed by 1 h incubation with the secondary antibody (1:5000) and detection by digital fluorescence imaging (LI-COR Odyssey CLx, Dallas, TX, United States). β-actin was used as the housekeeping gene. Densitometry of the bands was performed with ImageJ v.1.8.0 (http://imagej.nih.gov/ij (last access on 15 March 2022); National Institutes of Health, Bethesda, MD, United States). For ERK1/2 and phospho-ERK1/2, both 44 and 42 kDa bands were quantified and then summed.

### 2.8 RNA isolation and quantitative real-time PCR

Lung tissue, stored in an RNA later solution, was homogenized in TRIzol followed by a cleaning step using the RNeasy Mini Kit. The purified RNA was transcribed into cDNA using the SuperScriptTM IV VILO Reverse transcriptase kit (Invitrogen, Carlsbad, CA, United States) and was analyzed by real-time qPCR with SYBR Green Master Mix on a StepOne Plus Real-Time PCR System (Applied Biosystems v.2.3). The results were evaluated using the standard curve method and were expressed as the fold of the control values, normalized to β-actin. Specifically designed primer pairs and qPCR conditions were applied to selectively determine the expression of mouse *β-actin*, *Fibronectin*, *Collagen 1α2*, and *Elastin*, as previously described (Colunga Biancatelli et al., 2021a; Solopov et al., 2021).

### 2.9 Lung mechanics measurements

The mice were anesthetized with Socumb (pentobarbital sodium 50 mg/kg, i. p.), tracheostomized with a metal 1.2 mm (internal diameter) cannula, and connected to a FlexiVent ventilator (SCIREQ Inc., Montreal, QC, Canada), as previously published (Colunga Biancatelli et al., 2021a). Ventilation was performed at a tidal volume of 10 ml/kg and a respiratory rate of 150/min. Firstly, following a deep inflation, pressure volume (PV) loops were estimated by stepwise increasing airway pressure to 30 cm H_2_O and then reversing the process. PV loops reflect the intrinsic elasticity of the lungs and shifted to the right in fibrosis. Secondly, Snapshot-150 and Quick Prime-3 maneuvers were performed. Increasing concentrations of methacholine (to 50 mg/ml) were loaded into the nebulizer and administered to mice. Respiratory system resistance (Rrs) and elastance (Ers), reflecting the behavior of the entire respiratory system (peripheral and conducting airways, chest wall, and parenchyma); Tissue damping (G), reflecting resistance of the large, conducting airways, parenchymal stiffness, and changes in inspiratory gas dynamics, was calculated, and are presented as the mean of 12 recordings for each animal.

### 2.10 Statistical analysis

The results are presented as means ± standard error of the mean. Statistical significance of differences between groups was determined by the one- or two-way ANOVA analysis, followed by the Tukey’s or Bonferroni’s post-hoc test, utilized GraphPad Prism v. 9.0 (GraphPad, San Diego, CA, United States). The difference among groups was considered significant at *p* < 0.05.

## 3 Results

### 3.1 Estimation of the optimal therapeutic dose of the HSP90 inhibitor, TAS-116, against pulmonary fibrosis

**FIGURE 1:**
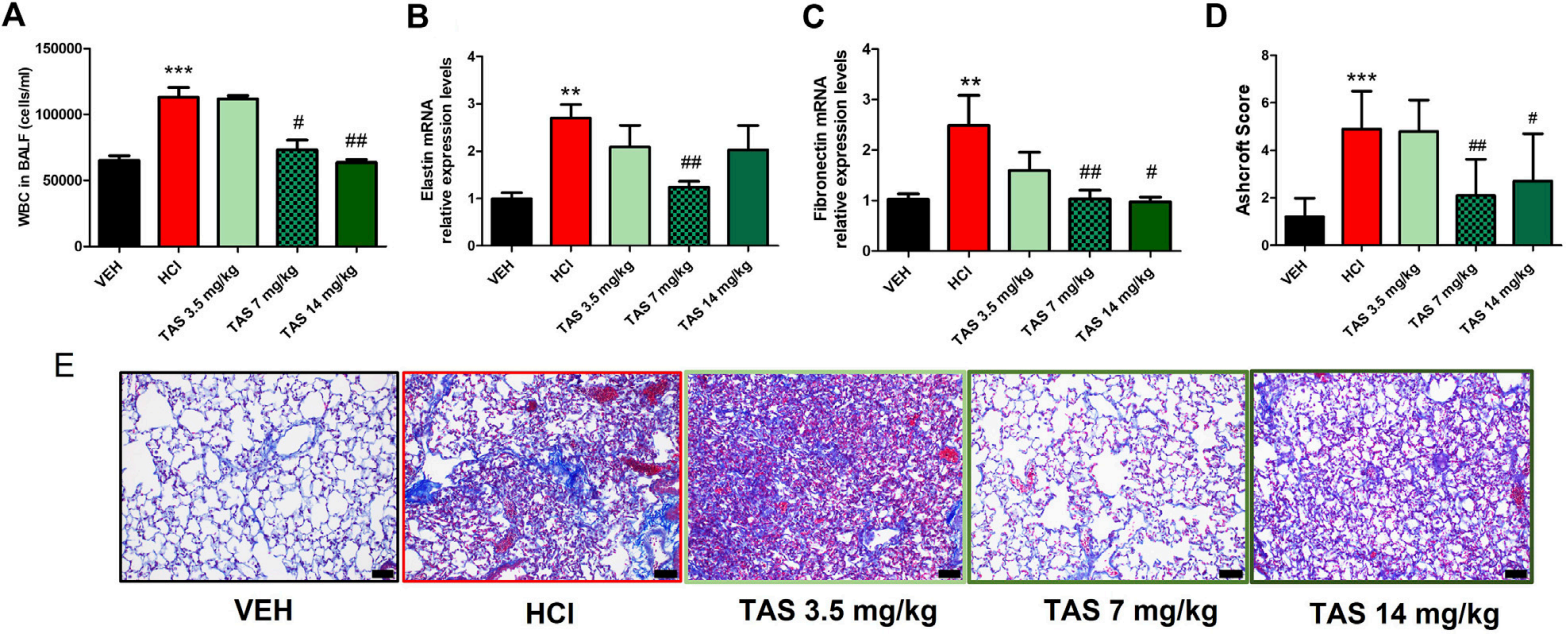
Analysis of lung injury after intratracheal instillaton of 0.1N HCl or saline followed by treatment, starting 24 h later, with the HSP90 inhibitor TAS-116 (3.5, 7 or 14 mg/kg 5×/week p. o.) for 30 days. Content of white blood cells in bronchoalveolar lavage fluid (BALF) *n =* 6 **(A)**; relative expression levels of Elastin **(B)** and Fibronectin **(C)** mRNA were analyzed by RT-PCR, *n = 4–5*; Ashcroft Score **(D)** and Masson’s Trichrome staining **(E)** of lung sections, *n = 3*. Original magnification ×20; black scale bars correspond to 50 µm; ^*^: *p* < 0.05; ^**^: *p* < 0.01, ^***^: *p* < 0.01, from VEH; ^#^: *p* < 0.05, ^##^: *p* < 0.01 from HCl, with 1-way ANOVA and Tukey’s post-hoc test.

First, we determined the effective therapeutic dose of TAS-116 as a protective agent against chronic lung injury and pulmonary fibrosis. Three different doses (3.5 mg/kg, 7 mg/kg and 14 mg/kg) were administrated *per os* 5 times/week to 0.1N HCl-challenged mice, starting 24 h after HCl; analyses were performed 30 days post HCl. Mice, instilled with HCl and treated with the inhibitor in the dose 3.5 mg/kg did not show significant improvement in any of the parameters measured (BALF cellularity, lung elastin and fibronectin mRNA levels, histology, Ashcroft score) compared to untreated mice (Figure 1). Both higher doses demonstrated significant decrease of leucocyte levels in BALF, deposition of fibronectin and improvement of parenchymal architecture in lung tissue (Figures 1A,C–E). However, only mice, treated with the dose 7 mg/kg showed decrease of Elastin (Figure 1B). The data obtained served as the basis for using the dose 7 mg/kg as the most effective for subsequent studies.

### 3.2 Delayed treatment with the HSP90 inhibitor, TAS-116

#### 3.2.1 Delayed treatment with TAS-116 reduces HCl-Induced chronic alveolar inflammation

**FIGURE 2:**
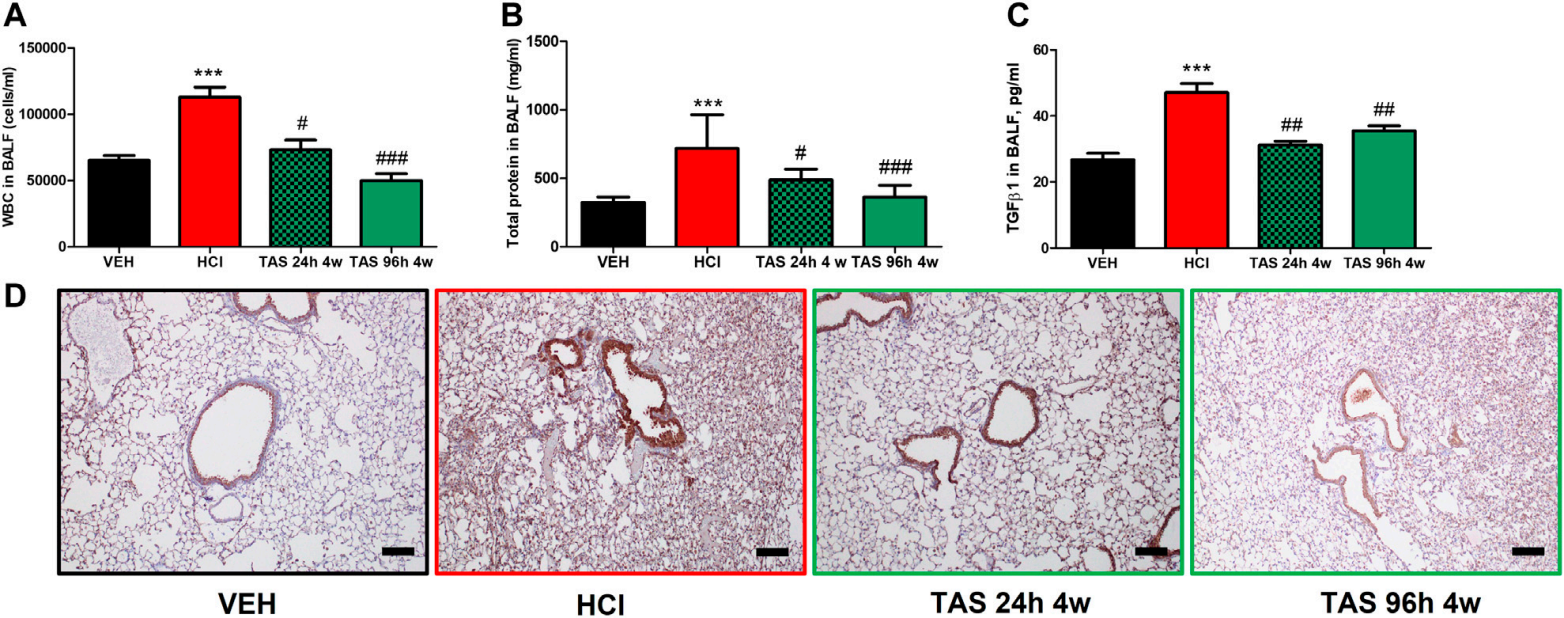
Oral treatment with TAS-116—starting either 24 or 96°h after HCl-ameliorates alveolar inflammation. **(A)** White blood cells, **(B)** Total protein concentration and **(C)** TGF-β content in bronchoalveolar lavage fluid (BALF) at 30°days post HCl instillation and treatment with TAS-116 at 7 mg/kg 5×/week starting 24 or 96 h post-HCl; **(D)** Immunohistochemical staining for the inflammasome NLRP3. Original magnification ×10; black scale bars correspond to 100 μm; *n* = 6 mice per group; ^***^: *p* < 0.01, from VEH; ^#^: *p* < 0.05, ^##^: *p* < 0.01, ^###^: *p* < 0.001 from HCl, with 1-way ANOVA and Tukey’s post-hoc test.

To determine the antidotal effectiveness of delayed treatment with TAS-116, we studied groups of HCl-instilled mice that started receiving the inhibitor 24 h or 96 h post HCl instillation. Leucocyte content and total protein concentration in BALF increased at 30 days post HCl; treatment with TAS-116, 7 mg/kg, starting 24 h after HCl, successfully blocked these increases (Figures 2A,B). The expression of TGF-β in BALF (Figure 2C) and NLRP3 inflammasome (Figure 2D) were also restored to control levels. Furthermore, mice who started receiving treatment 96 h after HCl instillation demonstrated equally effective antidotal activities.

#### 3.2.2 Delayed treatment with TAS-116 reduces hydrochloric acid-Induced pulmonary fibrosis

**FIGURE 3:**
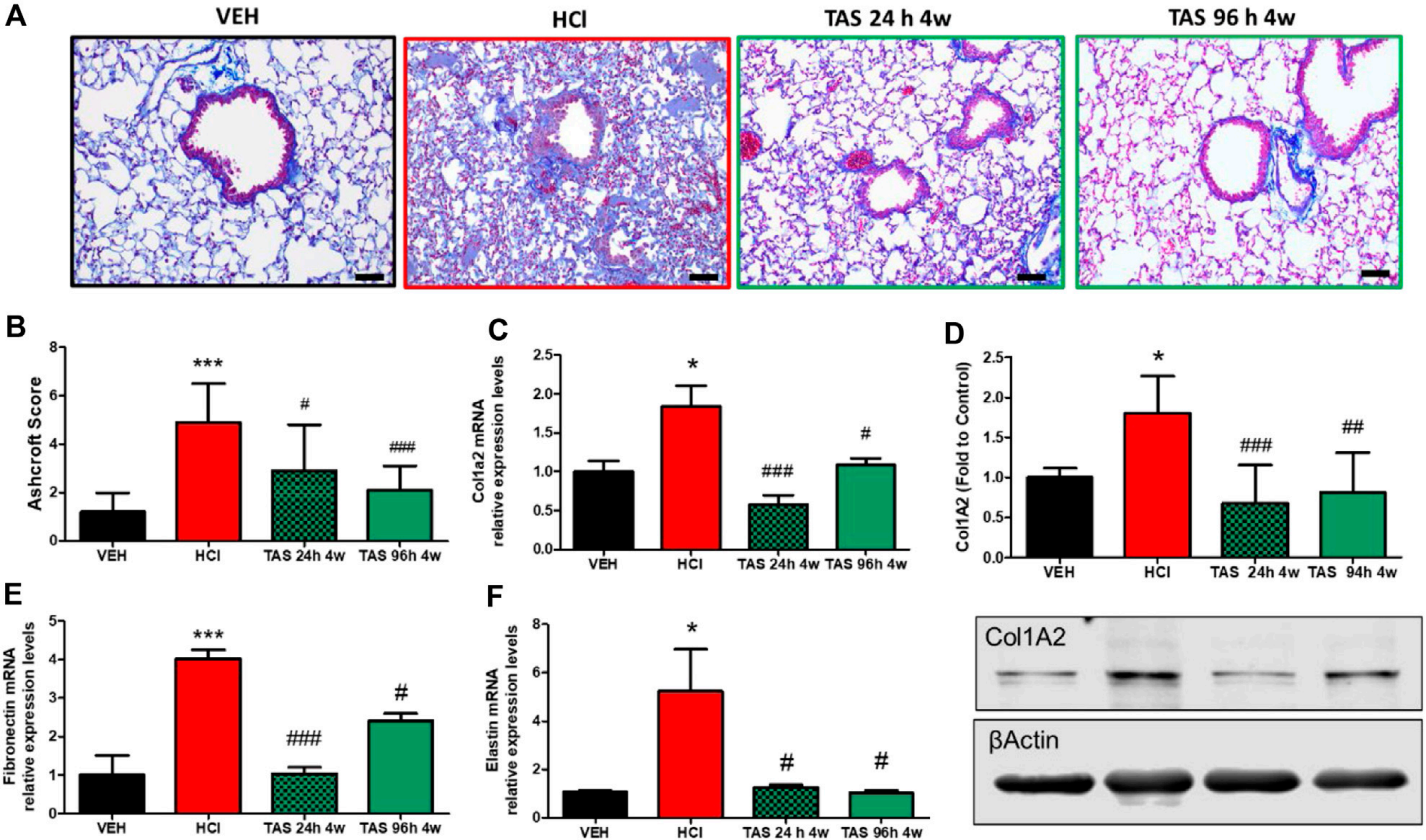
Oral treatment with TAS-116—starting either 24 or 96°h after HCl-ameliorates HCl-induced pulmonary fibrosis. Mice were intratracheally instilled with 0.1N HCl or saline followed by treatment, starting 24 or 96 h later, with the HSP90 inhibitor TAS-116 (7 mg/kg 5×/week p. o.) for 4°weeks. Masson’s Trichrome staining of lung sections demonstrates massive fibrous obliteration and loss of parenchymal architecture in HCl-instilled mice **(A)**. The Ashcroft score depicts severe fibrosis in HCl-instilled mice and recovered lungs in TAS-116-treated groups of mice **(B)**. The level of collagen type I was indicated *via* qPCR **(C)** and Western Blotting **(D)**. mRNA expression of extracellular matrix Fibronectin **(E)** and Elastin **(F)** were measured *via* qPCR. Original magnification ×20; black scale bars correspond to 50 µm (Means ± SEM; *n* = 4–6; ^*^: *p* < 0.05, ^***^: *p* < 0.01, from VEH; ^#^: *p* < 0.05, ^##^: *p* < 0.01, ###: *p* < 0.001 from HCl, with one-way ANOVA and Tukey’s.

Formalin-fixed lung sections were stained with Masson’s trichrome to visualize lung parenchymal changes and collagen deposition (Figure 3A). At 30 days after HCl instillation, a fibrotic process characterized by alveolar thickness and fibrous obliteration was observed. Mice receiving TAS-116, 7 mg/kg, 5 days/week, p. o., and starting either 24 h or 96 h after HCl, exhibited significantly decreased histopathologic changes and Ashcroft score values (Figure 3B). Overexpression of collagen Type I (Figures 3C,D), fibronectin (Figure 3E), and fibronectin (Figure 3F), analyzed by western blotting and real-time qPCR were also decreased in both treatment groups compared to the group instilled with HCl alone.

#### 3.2.3 TAS-116 Inhibits the activation of pro-fibrotic pathways

**FIGURE 4:**
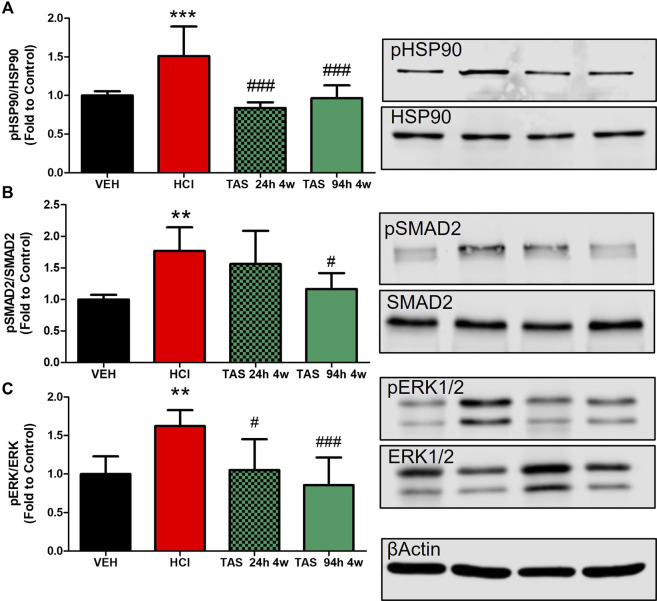
Oral treatment with TAS-116—starting either 24 or 96 h after HCl-ameliorates HCl-induced activation of pro-fibrotic pathways. **(A)** HSP90 activation (pHSP90) increased in HCl-instilled mice and was reduced in mice treated with TAS-116 24 h or 96 h post HCl. **(B)** Phosphorylated SMAD2 was significantly increased in mice instilled with HCl and reduced in group of mice, receiving treatment 96 h post instillation. **(C)** ERK1/2 (MAPK) activation (pERK) increased in HCl-instilled mice and reduced in both TAS-116-trated groups. Means ± SEM; ***: *p* < 0.001, **: *p* < 0.01, *: *p* < 0.05 from VEH; ^#^: *p* < 0.05, ^###^: *p* < 0.001 from HCl, with one-way ANOVA and Tukey’s, *n* = 6–7. VEH: Vehicle.

We analyzed the ability of TAS-116 to inhibit the expression of canonical and noncanonical pro-fibrotic signaling biomarkers: the activated forms of SMAD2 (mothers against decapentaplegic homolog 2), extracellular signal-regulated kinase (MAPK/ERK) and HSP90 (Figure 4). As we previously reported, instillation of hydrochloric acid increases the phosphorylation of SMAD2 and ERK1/2, and activates HSP90, at 30 days post-exposure. TAS-116, 7 mg/kg, 5x/week, starting 24 h after HCl did not significantly reduce pSMAD2 expression, however, treatment starting 96 h post HCl significantly reduced pSMAD2 expression when compared to HCl-instilled mice. At 30°days post-exposure, both TAS-116 treatment plans successfully blocked ERK and HSP90 activation, when compared to HCl-instilled mice.

#### 3.2.4 TAS-116 inhibits lung Dysfunction and airway hyper-responsiveness to methacholine

**FIGURE 5:**
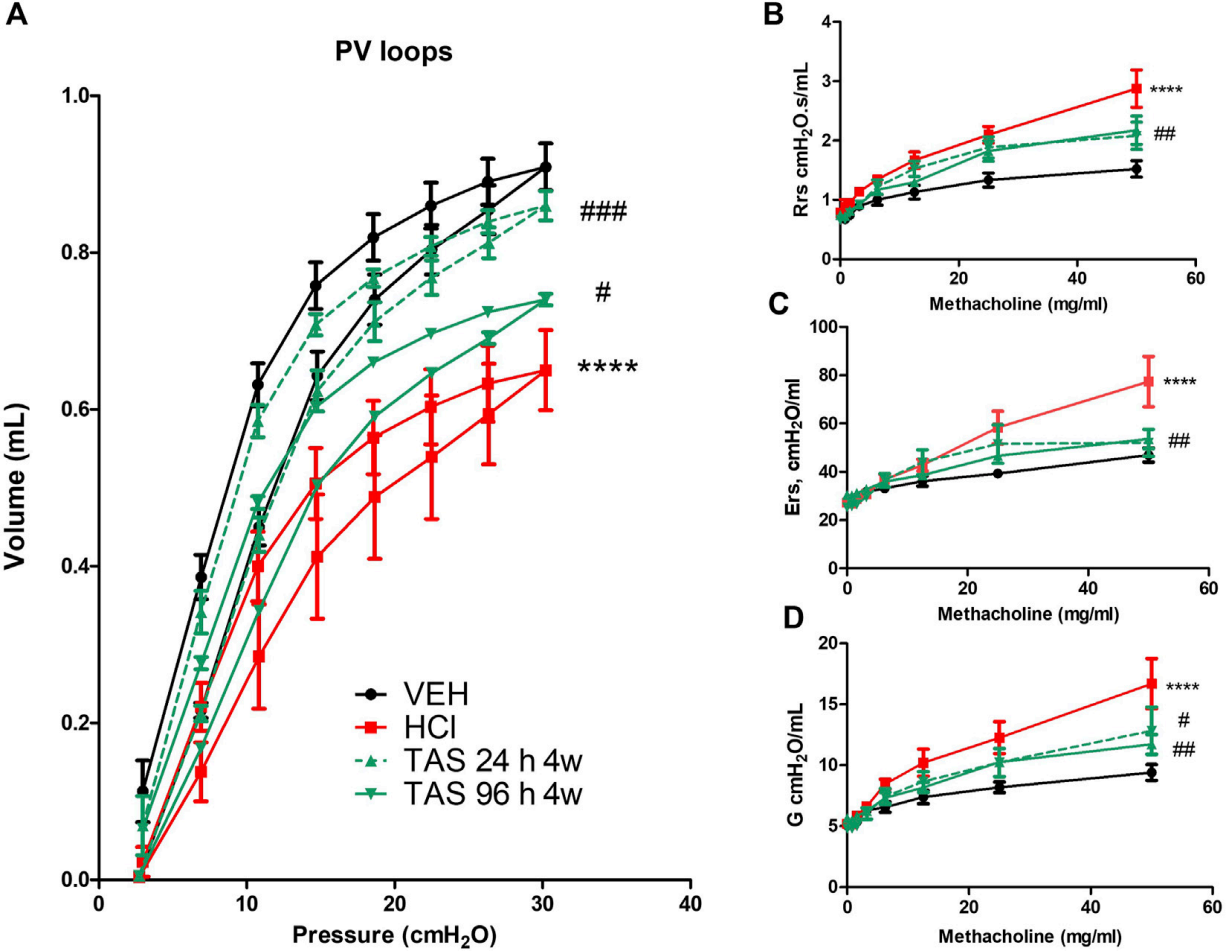
Oral treatment with TAS-116—starting either 24 or 96 h after HCl-ameliorates HCl-induced lung dysfunction. Both 24 h and 96 h treatment groups had a significant impact on preventing a downward shift of pressure volume (PV) loops after HCl instillation **(A)**. Respiratory system resistance (Rrs), elastance (Ers), and damping (G) increased, compared to control mice in all groups instilled with HCl **(B–D)**. This increase was significantly dampened in TAS-116 treated mice. Means ± SEM; *n* = 6 mice per group; ****: *p* < 0.0001 from VEH; ^#^: *p* < 0.05, ^##^: *p* < 0.01, ^###^: *p* < 0.001 from HCl, with one- or two-way ANOVA and Tukey’s post-hoc test.

Changes in lung mechanics were tested 30 days after acid instillation. Animals challenged with HCl demonstrated a downward shift in pressure-volume (PV) loops compared to VEH mice (Figure 5A). Both groups treated with 7 mg/kg TAS-116 displayed significant improvement, but treatment that started 24 h after HCl exposure brought the loop to the physiological norm. Total respiratory system resistance (Rrs), elastance (Ers), and tissue damping (G), increased in response to rising concentrations of methacholine in HCl-instilled mice (Figures 5B-D). Both TAS-116 treatments were equally able to prevent increases in those parameters.

### 3.3 Shortened treatment time with the HSP90 inhibitor TAS-116

#### 3.3.1 3 but not 2 weeks treatment with TAS-116 reduces HCl-induced chronic alveolar inflammation

**FIGURE 6:**
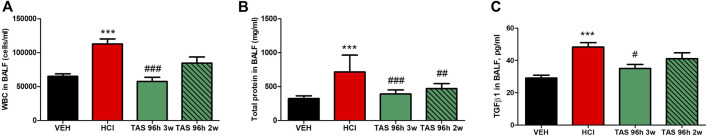
3 weeks treatment with TAS-116 ameliorates HCl-induced alveolar inflammation. **(A)** White blood cells, **(B)** total protein and **(C)** TGF-β concentrations in bronchoalveolar lavage fluid (BALF) at 30 days post HCl instillation and treatment with TAS-116, 7 mg/kg 5×/week, po, starting 96 h post-HCl; *n* = 6 mice per group; ***: *p* < 0.001 from VEH; ^#^: *p* < 0.05, ^##^: *p* < 0.01, ^###^: *p* < 0.001 from HCl; with 1-way ANOVA and Tukey’s post-test.

Having ascertained that 96 h delayed treatment with the HSP90 inhibitor TAS-116 can successfully reduce lung injury, we then reduced treatment time from 4 to 3 or to 2 weeks, to define the shortest and most effective treatment plan. Bronchoalveolar lavage fluid samples were analyzed for alveolar WBC recruitment, total protein concentration and overexpression of the key pro-fibrotic cytokine TGFβ. Mice treated for 14 days, starting 96 h post HCl instillation, demonstrated reduction of only total protein concentration in BALF, while 3 weeks treatment significantly ameliorated WBC, protein and TGFβ content (Figure 6).

#### 3.3.2 3 weeks treatment with TAS-116 reduces HCl-induced pulmonary fibrosis

**FIGURE 7:**
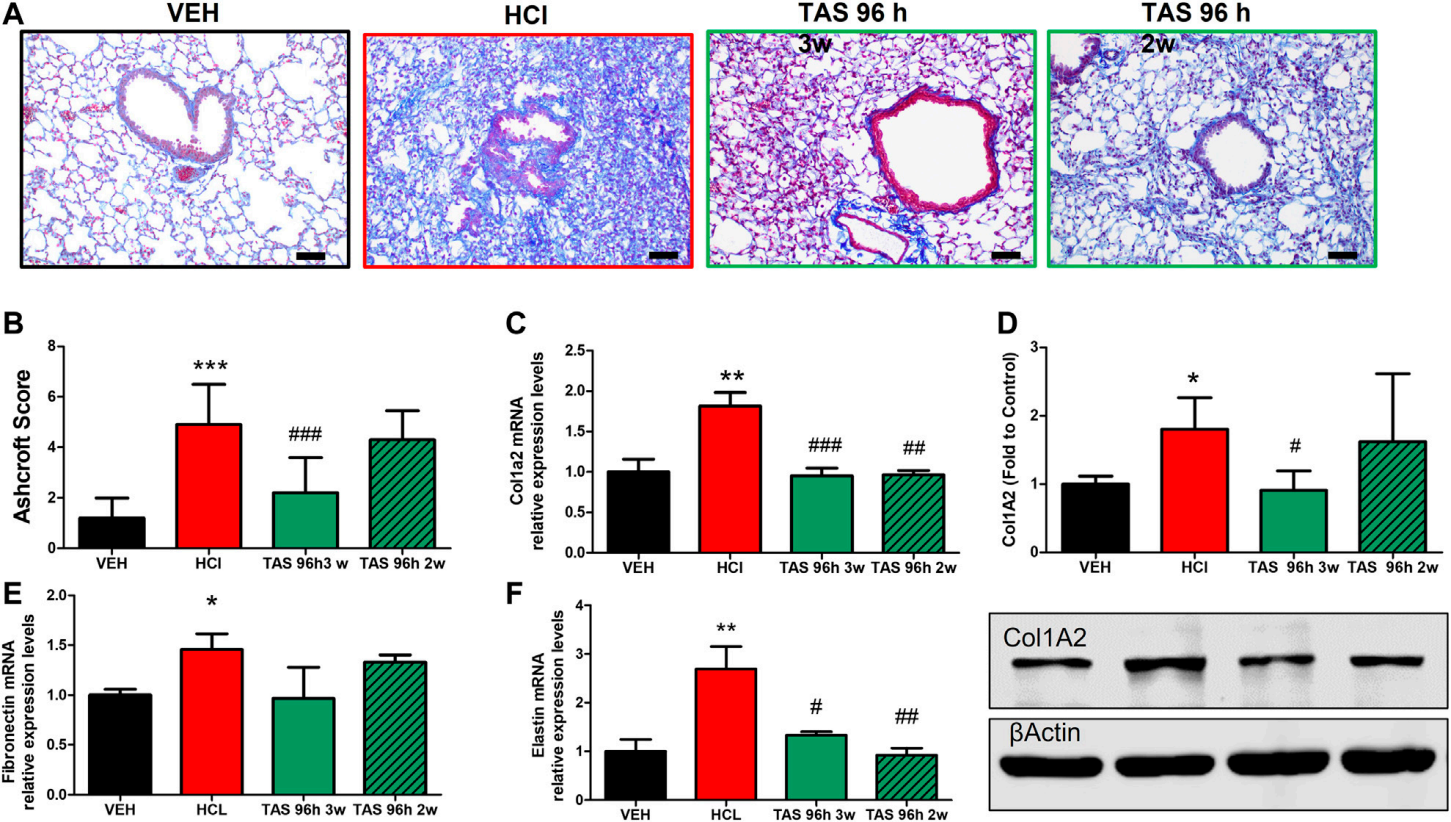
3 weeks treatment with TAS-116 ameliorates HCl-induced pulmonary fibrosis. Mice received intratracheal 0.1N HCl or saline followed by treatment, starting 96 h later, with TAS-116 (7 mg/kg 5×/week p. o.) for 2 or 3 weeks. Masson’s Trichrome staining of lung sections demonstrates accumulation interstitial, peribronchial and perivascular collagen in HCl-instilled mice. Animals treated with TAS-116 for 3 weeks exhibited improved lung architecture **(A)** and lower Ashcroft score **(B)**. The level of collagen type I was indicated *via* qPCR **(C)** and Western Blotting **(D)**. mRNA expression of extracellular matrix Fibronectin **(E)** and Elastin **(F)** were measured *via* qPCR. Original magnification ×20; black scale bars correspond to 50 µm (Means ± SEM; *n* = 4–6; ***: *p* < 0.001; **: *p* < 0.01, *: *p* < 0.05 from VEH; ^#^: *p* < 0.05, ^##^: *p* < 0.01, ^###^: *p* < 0.001 from HCl, with one-way ANOVA and Tukey’s post-hoc test.

Mice exposed to 0.1N HCl exhibited interstitial, peribronchial and perivascular accumulation of collagen and overexpression of ECM (Figure 7). Treatment with TAS-116 reduced by 2 weeks showed the improvement in collagen and elastin (Figures 7C,D,F), while 3 weeks-administration of inhibitor significantly improved histological signs of fibrosis (Figures 7A,B). The expression of fibronectin was not reduced by either treatment group (Figure 7E).

#### 3.3.3 3 weeks treatment with TAS-116 inhibits the activation of pro-fibrotic pathways

**FIGURE 8:**
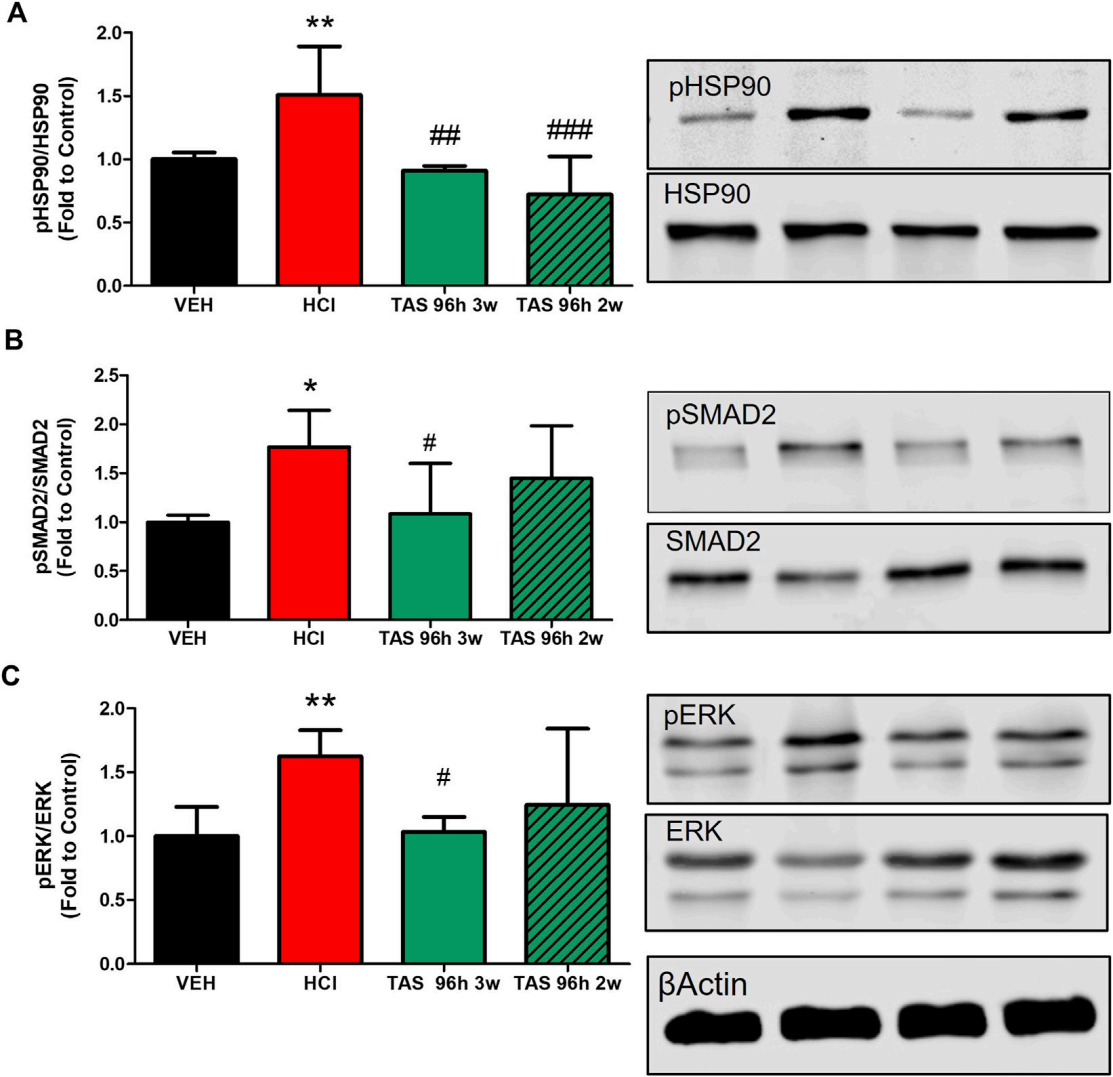
3 weeks treatment with TAS-116 ameliorates HCl-induced activation of pro-fibrotic pathways. **(A)** Heat Shock Protein 90 activation (pHSP90) increased in HCl-instilled mice and was reduced in mice, treated for 2 or 3 weeks with TAS-116, starting 96 h post HCl. **(B)** Active (phosphorylated) SMAD2 and **(C)** ERK1/2 were significantly increased in mice instilled with HCl and reduced in mice, treated for 3, but not 2 weeks with TAS-116. Means ± SEM; **: *p* < 0.01, *: *p* < 0.05 from VEH; ^#^: *p* < 0.05, ^##^: *p* < 0.01, ^###^: *p* < 0.001 from HCl, with one-way ANOVA and Tukey’s post-hoc test, n = 5–7.

Pro-fibrotic pathways were activated by HCl (Figure 8). Activation of HSP90 was significantly blocked in both 3- and 2-week TAS-116 treated groups. However, significant decrease in phosphorylation of SMAD2 and ERK1/2 was observed only in lung homogenates taken from mice after 3 weeks treatment with the HSP90 inhibitor.

#### 3.3.4 3 weeks treatment with TAS-116 improves HCl-induced lung dysfunction and airway hyper-responsiveness to methacholine

**FIGURE 9:**
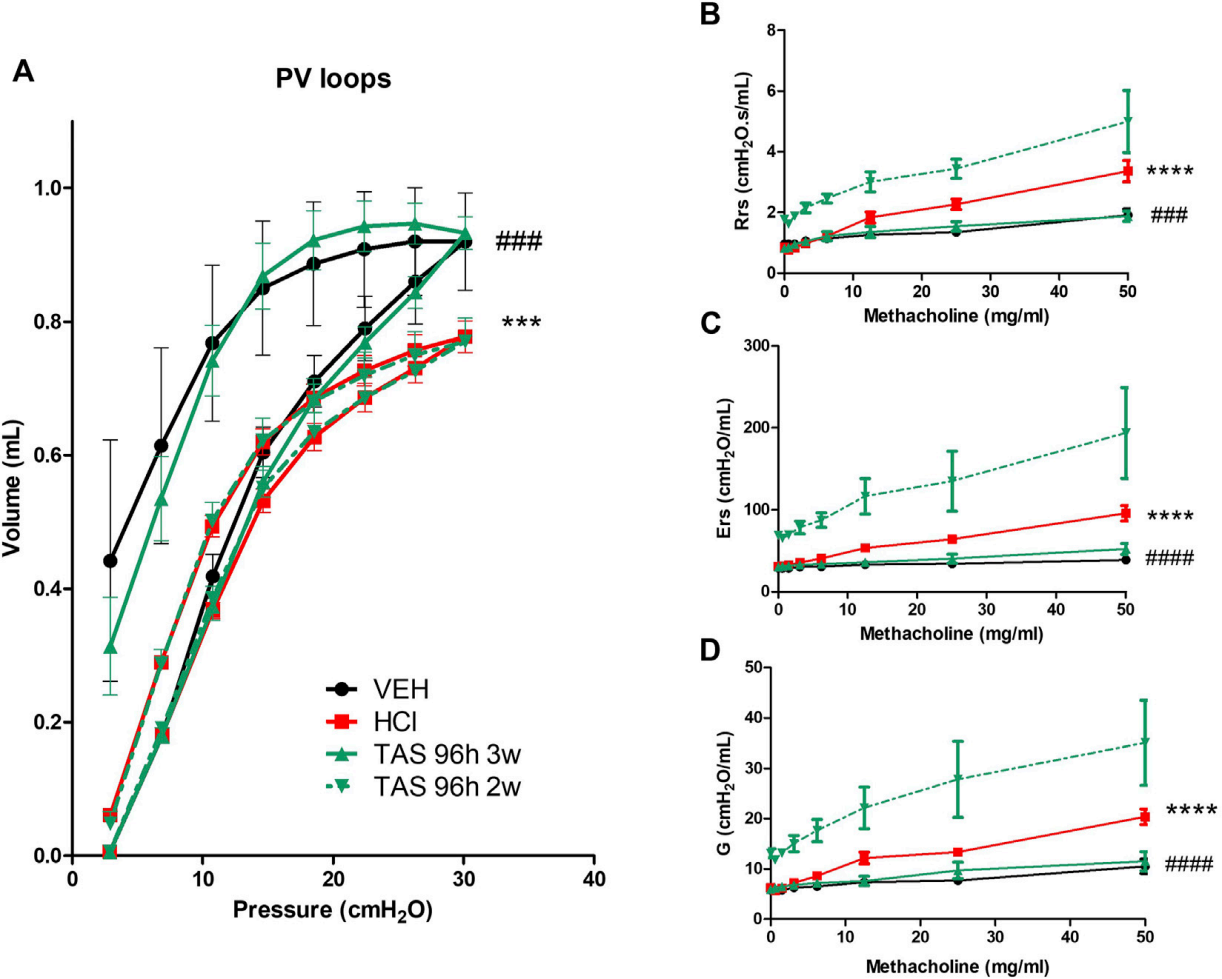
3 weeks treatment with TAS-116 ameliorates HCl-induced lung dysfunction and airway hyper-reactivity. **(A)** Pressure–Volume relationships **(B)** Total Respiratory System Resistance (Rrs), **(C)** total Respiratory System Elastance (Ers), and **(D)** tissue damping (G), All studies were performed at 30 days after HCl instillation. *n* = 4–6 mice per group; ***: *p* < 0.001, ****: *p* < 0.0001 from VEH; ^###^: *p* < 0.001, ^####^: *p* < 0.0001 from HCl, with 2-way ANOVA and Bonferroni’s post-hoc test.

HCl-challenged animals showed a decrease in pressure-volume (PV) loops (Figure 9A). Mice, treated with 7 mg/kg TAS-116 for 3 weeks displayed complete recovery, in contrast to mice treated for 2 weeks that showed no improvement. Total respiratory system resistance (Rrs), elastance (Ers), and tissue damping (G), were increased in response to methacholine in HCl-treated mice; this was completely prevented by 3-week, but not 2-week treatment with TAS-116 (Figures 9B–D).

## 4 Discussion

In this study we used TAS-116, a novel class of selective inhibitors of heat shock protein 90, as a potential antidote against chronic lung injury and pulmonary fibrosis, caused by intratracheal exposure to hydrochloric acid. We selected this inhibitor based on the following features. Unlike past generation-inhibitors, TAS-116 targets cytosolic HSP90α and HSP90β, without affecting HSP90 in the mitochondria and endoplasmic reticulum (Ohkubo et al., 2015). HSP90, a causative factor in pulmonary fibrosis, was localized in both the cytosol and nucleus of fibroblasts (Sontake et al., 2017). Additionally, TAS-116 has oral bioavailability, which allows administration on an outpatient basis. Further, this drug has already shown its safety in a randomized, double-blind, Phase III clinical trial comparing TAS-116 to placebo in patients with gastrointestinal stromal tumor refractory to standard treatments (Honma et al., 2021).

Our first aim was to find an effective and safe dose of TAS-116 for mice against HCl-induced pulmonary fibrosis. In various clinical trials against cancer, this compound was used in doses of 80–160 mg on a 5 days on/2 days off regimen (Kurokawa et al., 2017; Kawazoe et al., 2021). In animal studies, the dose 14 mg/kg was considered as safe and effective for mice against tumors with the same administration schedule (Saito et al., 2020). In this study, we used three different doses in search of the minimal effective dose against PF. Mice, receiving 3.5 mg/kg starting 24 h after HCl instillation did not show significant improvements compare to untreated HCl-challenged mice, while mice receiving either 7 or 14 mg/kg of inhibitor demonstrated antidotal effects. Based on that, we considered that TAS-116, at 7 mg/kg, as the optimal therapeutic dose for mice.

Despite studies suggesting that the pathophysiology of pulmonary fibrosis is a product of fibroblast dysfunction, inflammation remains one of the critical factors in the disease. Patients with IPF have periods of exacerbations or acute decline. During exacerbations, the recruitment of inflammatory cells is seen in BALF with numerous proinflammatory mediators, cytokines, and growth factors (Parambil et al., 2005; Kim et al., 2006; Bringardner et al., 2008). We previously demonstrated that the peak of acute lung injury in mice occurs on the fourth day after HCl instillation but that low-to-moderate inflammation persists through day 30 (Marinova et al., 2019). The involvement of pro-inflammatory pathways in the development of PF has also been demonstrated in various mouse models (Izbicki et al., 2002; Marinova et al., 2019; Solopov et al., 2020). Several publications further suggest that the NLRP3 inflammasome plays an important role in fibrogenesis (Ma et al., 2020; Ranta-aho et al., 2021; Colunga Biancatelli et al., 2022b). Here, we analyzed the leucocyte and total protein contents in bronchoalveolar lavage, the expression of the pro-inflammatory and pro-fibrotic cytokine, TGF-β, and of NLRP3 inflammasome, which designate inflammation of alveoli, migration of immune cell, and alveolar-capillary hyper-permeability. All were significantly exacerbated by HCl, in agreement with previous studies by us and others. We previously reported the ability of HSP90 inhibitors (AT13387 and AUY-922) to modulate human pulmonary microvascular endothelial activation and barrier dysfunction, which is responsible for vascular permeability and exudation of inflammation products into the alveolar space (Colunga Biancatelli et al., 2021b; Colunga Biancatelli et al., 2022c). Treatment with HSP90 inhibitor TAS-116 in dose 7 mg/kg starting either 24 or 96 h post-HCl successfully ameliorated all signs of inflammation even with a shortened, 3 weeks treatment course.

Fibrosis is traditionally associated with pathological changes in the extracellular matrix. We have repeatedly reported that a single exposure to 0.1N hydrochloric acid leads to pulmonary fibrosis in mice, which is reflected in upregulation of ECM, and have considered HSP90 as a potential therapeutic target (Marinova et al., 2019; Solopov et al., 2020; Colunga Biancatelli et al., 2021a; Solopov et al., 2021). The expression of ECM proteins (fibronectin, elastin) varied in magnitude among studies (e.g., experiments shown in Figure 3 vs. Figure 7) but was always elevated at 30 days after HCl. The effectiveness of HSP90 inhibitors in modulating ECM secretion has been shown in several studies. HSP90 chaperones cannot directly engage nascent molecules of the matrix, however, there are indirect mechanism though which HSP90 inhibitors block overexpression of ECM. The HSP90α inhibitor 17-allylaminogeldanamycin (17-AAG) tightly decreases collagen-I expression in human primary cells (Wong et al., 2018). The exogenous beta isoform of Hsp90 activates the formation of extracellular fibronectin in breast cancer cells, while knockdown or inhibition of Hsp90β leads to reduced deposition and can be partially rescued by the addition of exogenous Hsp90 (Hunter et al., 2014). As we previously reported, HSP90 inhibitors AUY-922 (Marinova et al., 2020) and AT-13383 (Colunga Biancatelli et al., 2022a) significantly downregulate the overexpression of collagen, fibronectin and elastin expression in mice with HCl-induced pulmonary fibrosis. In the present study, we observed the formation of interstitial, peribronchial and perivascular collagen masses that significantly changed lung architecture. Both 24 h- and delayed treatment with TAS-116 decreased collagen deposition and overexpression of fibronectin and elastin.

The cytoplasmic proteins SMAD2/3 mediate signals from activated TGF-β1 receptors. Phosphorylation of SMAD2/3 allows their binding to SMAD4, promoting translocation to the nucleus where numerous TGF-β1-responsive genes are activated. Hsp90 plays an important role in inflammatory processes by stabilizing and activating more than 300 ‘client’ proteins, including key proinflammatory signaling molecules, such as nuclear transcription factors (e.g., NF-κB, STATs, p53) and kinases (e.g., Raf/MEK/ERK, PI3K/AKT, p38/MAPK) (Trepel et al., 2010). Activation of Hsp90 reflected in HSP90 deacetylation and tyrosine phosphorylation (Dimitropoulou et al., 2013). Heat shock protein 90 actively participates in the TGF-β signaling pathway and HSP90 inhibition reduces fibrogenesis and lung fibrosis progression in mice (Bonniaud et al., 2017). Sontake et al. found elevated HSP90 immunostaining in lung biopsy samples of patients with IPF. Moreover, the suppression of HSP90 by another inhibitor 17-AAG moderated migration of myofibroblasts and, as a result, ECM production. (Sontake et al., 2017). One of the client proteins of HSP90 is mitogen-activated protein kinase (MAPK). MAPK kinase (MEK)/extracellular signal–regulated kinase (ERK) is also involved in fibrogenesis *via* growth and proliferation. Activation of the ERK1/2 has been detected in human fibrotic lungs (Yoshida et al., 2002; Antoniou et al., 2010). Overexpression of TGF-α in lung epithelium of transgenic CCSP/TGF-α mice causes progressive lung fibrosis and inhibition of ERK prevents the progression (Le Cras et al., 2010). In gastrointestinal stromal tumors, TAS-116 inhibits ERK1/2 (Saito et al., 2020). We previously reported that HSP90 inhibitors AUY-922 and AT13387 reduce both SMAD2 and ERK phosphorylation *via* blocking the activation of HSP90 in mice treated with HCl. (Marinova et al., 2020; Colunga Biancatelli et al., 2022a). Here we show that oral treatment with TAS-116 exerts similar therapeutic effects.

Development of pulmonary fibrosis affects not just alveoli but also conducting airways and the lung vasculature. Patients with IPF show alterations in lung dynamics (Plantier et al., 2018). The instillation of HCl in mice provoked strong changes in lung mechanics such as elevated total Respiratory System Elastance, Respiratory System Resistance, and tissue damping. Furthermore, HCl shifted down pressure–volume loops. Mice exposed to HCl and treated with TAS-116 successfully restored systemic resistance, elastance, damping, and corrected the downward shift of PV loops.

In conclusion, we demonstrated that TAS-116 prevents the development of HCl-induced chronic lung injury and pulmonary fibrosis by blocking the overexpression of pro-fibrotic markers, reducing collagen deposition and maintaining lung dynamics within physiological ranges. Moreover, this HSP90 inhibitor is effective even when treatment begins at the peak of acute lung injury, 96 h post HCl exposure, and lasts for 3 weeks. These benefits of TAS-116, coupled with oral bioavalibility, suggest that it may be a promising countermeasure against HCl-induced pulmonary fibrosis.

## Data Availability

The raw data supporting the conclusions of this article will be made available by the authors, without undue reservation.
